# Comparison of cell-free and small extracellular-vesicle-associated DNA by sequencing plasma of lung cancer patients

**DOI:** 10.1016/j.isci.2024.110742

**Published:** 2024-08-14

**Authors:** Norbert Moldovan, Sandra Verkuijlen, Ymke van der Pol, Leontien Bosch, Jan R.T. van Weering, Idris Bahce, D. Michiel Pegtel, Florent Mouliere

**Affiliations:** 1 Amsterdam UMC, Vrije Universiteit Amsterdam, Department of Pathology, Cancer Center Amsterdam, 1081 HV Amsterdam, the Netherlands; 2 Amsterdam UMC, Vrije Universiteit Amsterdam, Department of Human Genetics and Functional Genomics, Center for Neurogenomics and Cognitive Research, 1081 HV Amsterdam, the Netherlands; 3 Amsterdam UMC, Vrije Universiteit Amsterdam, Department of Pulmonology, Cancer Center Amsterdam, 1081 HV Amsterdam, the Netherlands; 4 Cancer Research UK National Biomarker Centre, University of Manchester, Manchester, UK

**Keywords:** Molecular genetics, Cancer systems biology, Cancer

## Abstract

Blood contains multiple analytes that can be used as liquid biopsy to analyze cancer. Mutations have been detected in DNA associated with small extracellular vesicles (sEVs). The genome-wide composition and structure of sEV DNA remains poorly characterized, and whether sEVs are enriched in tumor signal compared to cell-free DNA (cfDNA) is unclear. Here, using whole-genome sequencing from lung cancer patients we determined that the tumor fraction and heterogeneity are comparable between DNA associated with sEV (<200 nm) and matched plasma cfDNA. sEV DNA, obtained with size-exclusion chromatography, is composed of short ∼150–180 bp fragments and long >1000 bp fragments poor in tumor signal. The structural patterns of sEV DNA are related to plasma cfDNA. Mitochondrial DNA is relatively enriched in the sEV fractions. Our results suggest that DNA associated to sEV (including exosomes) is not preferentially enriched in tumor signal and is less abundant than cfDNA.

## Highlights

• Small extracellular vesicle (sEV) DNA genomic content remains poorly characterized
• sEV DNA and cell-free DNA (cfDNA) from plasma of 16 lung cancer patients were sequenced
• sEV DNA is ∼12 times less abundant per mL of plasma compared to cfDNA
• sEV DNA relative tumor fraction and heterogeneity is comparable to cfDNA

## Graphical abstract

**Figure:**
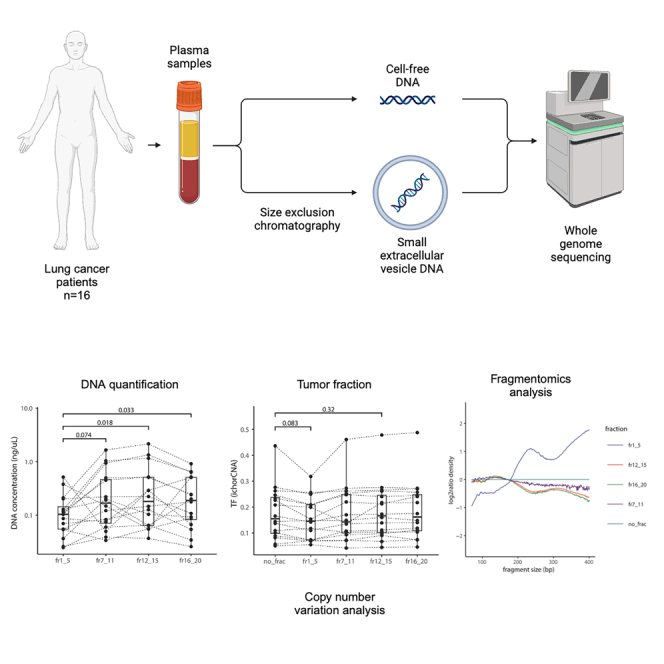

## Introduction

Liquid biopsy provides non-invasive ways to analyze genetic alterations, detect cancer early, or monitor residual disease in patients with cancer.^1^^,^^2^^,^^3^ Cell-free DNA (cfDNA) is the current gold standard for the analysis of genetic alterations with liquid biopsy, but this analyte has clear biological and technical limitations.^1^^,^^4^^,^^5^ cfDNA is thought to be released via cell-death mechanisms, the majority through apoptosis.^5^^,^^6^ cfDNA might therefore not be released by cancer cells in dormancy or with slowed proliferating cycle. Moreover, the genetic representation of cfDNA is biased toward shared clones and might underrepresent private mutations.^7^

Extracellular vesicles (EVs) could complement cfDNA as a liquid biopsy. Some EVs can be actively secreted by living cells,^8^ therefore potentially informing on cell populations underrepresented in cfDNA molecules.^9^^,^^10^ Due to this biological difference in their origin, the representation of tumor heterogeneity could differ between EV-DNA and plasma cfDNA.^7^ EVs have been reported to contain or be associated to DNA.^11^^,^^12^^,^^13^^,^^14^

Prior reports exhibit conflicting results and conclusions on whether EVs, in particular sEVs, contain tumor DNA signals. Approaches based on mutation testing (e.g., digital PCR) have identified either increased or decreased tumor fraction in DNA from sEV fraction.^14^^,^^15^^,^^16^^,^^17^ This is possibly a consequence of the different approaches employed to enrich for sEV and/or isolation of specific subpopulations. However, a focus on single loci and mutations in samples with low DNA concentration could be afflicted by high noise-to-signal ratio and increased stochastic noise in mutation signal. Thus, there is a need to evaluate the characteristics of EV-DNA using both sequencing and non-sequencing approaches and evaluate whether these molecules can be enriched in tumor DNA on a genome-wide scale.

Here, we determine the structure and genomic composition of DNA molecules in sEV fractions obtained by standardized size exclusion chromatography (SEC) and shallow whole-genome sequencing (WGS) and compare them to unfractionated plasma cfDNA from lung cancer patients. In particular, we compare copy-number aberrations, size profiles, composition in bases at the end of DNA fragments, and proportion in mitochondrial DNA between the different fractions.

## Results

### DNA in sEV fractions is composed of short and long fragments

**Figure 1:**
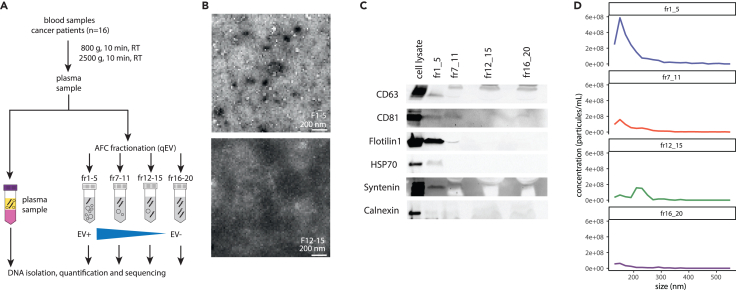
Experimental workflow and quality control of the EV separation (A) Schematic workflow of the AFC fractionation and DNA analysis. (B) Representative EM micrographs of the structures in fraction 1–5 (left panel) and fraction 12–15 (right panel). The white bar indicates the scale (200 nm). (C) Representative western blot with a range of EV-related targets for the different fractions. (D) Determination of the particle sizes (in nm) and concentration (in particles/mL) using a TRPS device depending on the AFC fractions.

To characterize tumor-derived DNA from sEV fractions, we collected plasma from 16 patients with lung cancer (Figure 1A; Table S1); 3 mL of plasma was aliquoted for cfDNA analysis using shallow WGS (sWGS) as previously described. We subjected plasma to SEC with the automated fraction collector (AFC) protocol as indicated by the manufacturer (see STAR methods). Electron microscopy (EM) revealed vesicle-like membrane structures that range mostly from 50 to 200 nm are enriched in AFC fractions 1–5 while seemingly absent in fractions 12–15 (Figures 1B and S1). Next, we implemented western blotting against known markers of small EV (including CD63 and CD81) and proteins (see STAR methods), confirming enrichment in small EV in fractions 1–5, and depletion in the other fractions (Figure 1C). Particle analysis using Exoid (Izon), based on tunable resistive pulse sensing (TRPS), confirmed that fractions 1–5 are enriched in small particles with a mean size distribution of ∼144 nm. The particle analysis matched the observation from the corresponding EM images (Figure 1D). Importantly, the concentration in small particles of this particular size range is progressively decreased in the other AFC fractions (7–11; 12–15; 16–20) (Figure 1D). These data show that plasma sEVs can be enriched in fractions 1–5 and relatively depleted in others using the AFC methodology.

**Figure 2:**
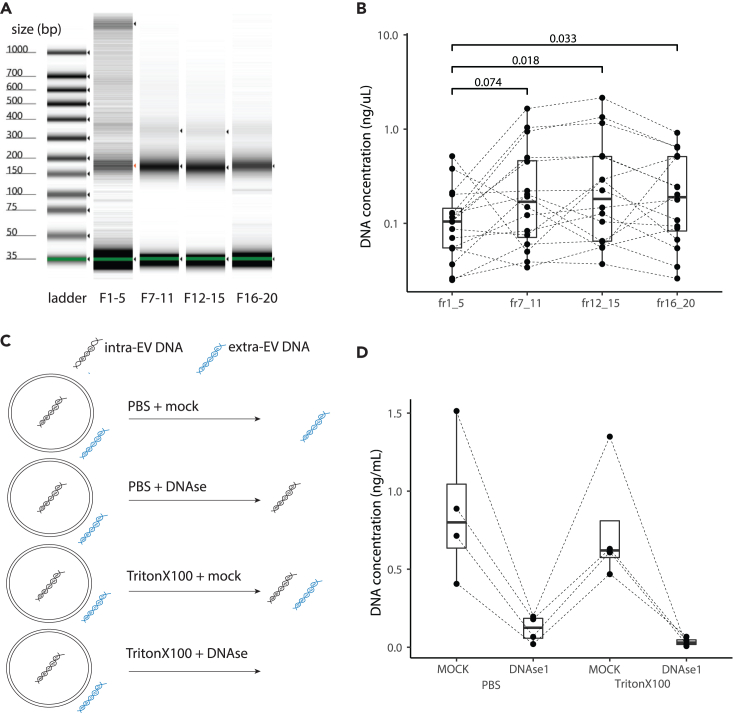
DNA concentration is low in EV fractions (A) Concentration (color intensity) and size (bp) of the DNA in the different fractions determined using an electrophoresis device (patient Code53 is shown in this plot). (B) Concentration in ng/uL of the DNA in the different AFC fractions determined using an electrophoresis device for all patients. *p* values are indicated (paired Wilcoxon test). (C) Schematic explaining the different treatments applied to the EV fractions from four samples. (D) Concentration in ng/uL of the DNA in the different fractions depending on the treatment conditions.

Automated gel electrophoresis indicates the presence of both short and long DNA in the AFC vesicle fractions 1–5 enriched in sEV but only short DNA in the other fractions (Figures 2A andS2). Despite this size difference, the observed concentration in DNA is significantly higher in fraction 12–15 and 16–20 compared to the fraction 1–5 (respectively *p* = 0.018 and *p* = 0.033, paired Wilcoxon test) (Figure 2B). The DNA concentration was not significantly different when comparing the fraction 1–5 to the fraction 7–11. To determine whether this DNA is compacted inside sEV or at their surface, we treated an aliquot from four patients with either DNase, a nonionic surfactant (Triton X-100) or a combination of both (Figure 2C, see STAR methods). Post-treatment, DNA was still detectable outside of sEVs, and the concentration was significantly lower within sEVs (Figure 2D).

### EV fractions are not enriched in tumor DNA

**Figure 3:**
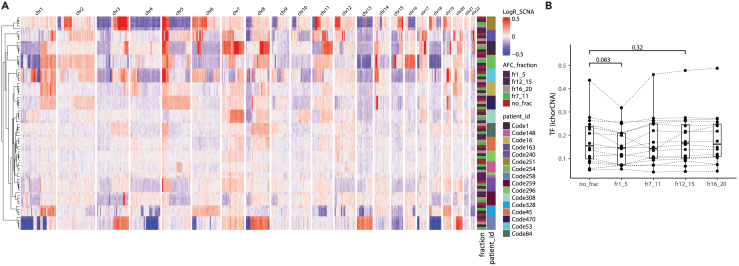
EV fractions are not enriched in tumor-derived DNA (A) Heatmap of the log_2_ratio copy-number aberrations calculated using sWGS data. Rows are samples, and columns are genomic positions (100 k bins). Columns are grouped depending on the chromosomes. Blue indicates a relative decrease in copy numbers and red an increase in copy numbers. The respective patients from which the samples are taken and the type of fractions are indicated as a left annotation. (B) Tumor fraction as estimated from the sWGS data using ichorCNA. *p* values are indicated (paired Wilcoxon test).

Recovering somatic copy-number aberrations could give a potentially more accurate genome-wide determination of the overall tumor fraction of plasma cfDNA samples in comparison to mutation analysis from limited loci.^18^ Using sWGS and the ichorCNA software, we determined the tumor fraction in our samples and observed no significant differences between the different AFC fractions (1–5, 7–11, 12–15, 16–20) and the non-fractionated cfDNA plasma sample (paired Wilcoxon test) (Figures 3A and 3B). Analysis of the difference in the amplitude of the log_2_ratio from the individual genomic bins reveals no difference between the non-fractionated cfDNA plasma samples and the different AFC fractions (Figure 3A). The different AFC fractions from the same patients cluster together with no apparent difference in the log_2_ratio of the copy-number aberrations detected, suggesting that the tumor genomic heterogeneity is represented in a similar fashion in cfDNA, and EV fractions (Figures 3A and S3). We also determined that the long DNA (>1,000 bp) identified in the sEV fractions (1–5) (Figure 2A) were exhibiting a significantly lower tumor fraction in comparison to the matched cfDNA from plasma samples or from the other fractions (Figure S4). Our results indicate that the AFC fractions 1–5, enriched in sEV, are not enriched in tumor-derived DNA in comparison to the other AFC fractions or matched unfractionated plasma cfDNA.

**Figure 4:**
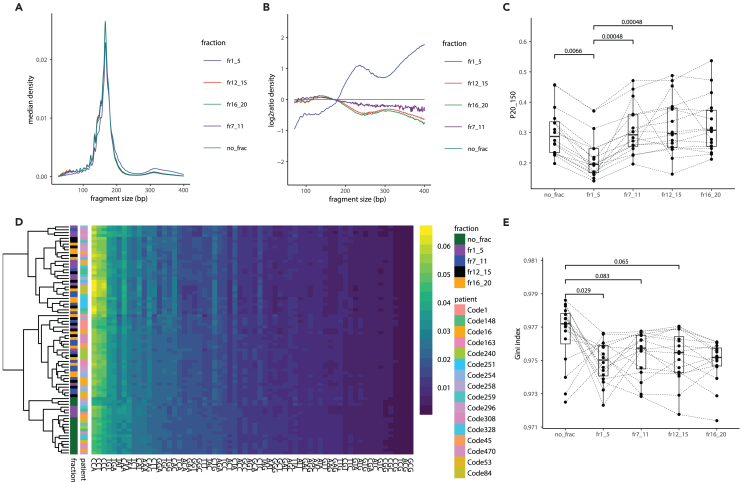
EV fractions are enriched in longer DNA fragments (A) Median DNA fragment sizes distribution for each of the AFC fractions and matched non-fractionated cfDNA samples. (B) Median log_2_ratio of the DNA fragment size distribution in comparison to the median size distribution of non-fractionated plasma cfDNA samples. (C) Proportion of DNA fragment between 20 and 150 bp (P20_150). *p* values are indicated (paired Wilcoxon test). (D) Heatmap of the proportion of trinucleotides at the end of DNA fragments from the different AFC fractions and matched cfDNA plasma samples. Yellow indicates high proportions and blue low proportions. The respective patients from whom the samples are taken and the type of fractions are indicated as a left annotation. (E) Diversity in the proportion of fragment-end trinucleotides calculated using a Gini index for the DNA of the AFC fractions and matched cfDNA plasma samples. *p* values are indicated (paired Wilcoxon test).

In addition to the copy-number aberrations, we analyzed the size of the DNA in the different fractions, as it was previously demonstrated this feature can reflect the presence of tumor-derived cfDNA in plasma.^19^ DNA size can be analyzed using the same paired-end sWGS data used for determining their copy-number aberrations. We observed in all four fractions (1–5, 7–11, 12–15, 16–20) a DNA size distribution with the typical mode at 167 bp previously observed for cfDNA (Figures 4A and S5). The longer DNA fragments are relatively enriched in the EV-rich fractions (1–5) in comparison to the non-fractionated plasma cfDNA and the other AFC fractions (Figures 4B and S6). The proportion of small DNA fragments between 20 and 150 bp (P20_150) are significantly higher in EV-poor fractions (7–11, 12–15, 16–20) than in the EV-rich fractions (1–5) (Figure 4C). Beyond the DNA fragmentation, the DNA fragment-end composition was retrieved from the same sequencing data. cfDNA fragment-end in plasma reflects the mechanisms and enzymatic cleavages, leading to their release in the circulation.^20^ The composition and diversity in trinucleotides at the 5′ end of the DNA fragments reveal no significant differences between the different AFC fractions but do highlight a difference between unfractionated cfDNA and AFC fractions (Figures 4D and 4E).

### Non-nuclear DNA are relatively enriched in sEV fractions

**Figure 5:**
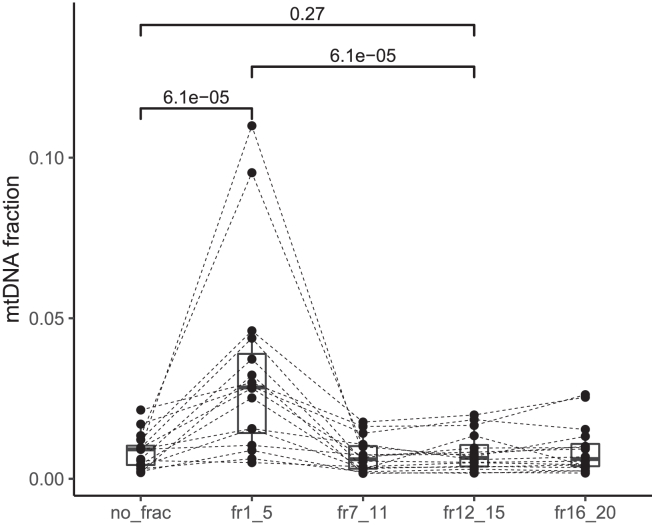
EV fractions are relatively enriched in mitochondrial DNA fragments The boxplots indicate the proportion of mitochondrial DNA fragments per fractions. *p* values are indicated (paired Wilcoxon test).

Beyond nuclear DNA, using untargeted sequencing like sWGS allowed to recover different types of DNA molecules in plasma.^21^^,^^22^ We reanalyzed the same dataset by estimating the presence of mitochondrial DNA in the non-fractionated and fractionated samples (Figure 5). The fraction of mitochondrial DNA remained a minor component of the total pool of plasma cfDNA (median = 0.69% of the total cfDNA pool). A significant increase in the proportion of mtDNA fragments can be observed in the sEV-rich fractions (1–5) (median = 3.1%, ∼5-fold relative enrichment) but not in the other fractions (7–11, 12–15, 16–20) (for fr1-5, *p* = 0.031, paired-Wilcoxon test, non-significant for other fractions). Due to their biological similarities with mitochondrial DNA, other non-nuclear DNA in plasma (e.g., bacterial DNA) are likely to be affected alike in sEV fractions.^21^^,^^23^

## Discussion

Our results indicate that DNA can be consistently detected in sEV fractions (1–5) from the plasma of lung cancer patients obtained with a standardized SEC procedure but equally present in fractions (7–11, 12–15, 16–20) with less detectable EVs. The presence of particles in the size range of small EVs (<200 nm) was confirmed by particle analysis and EM imaging. The biochemical properties (fragmentation pattern, size, fragment-end composition) of the DNA identified in the EV-poor fractions are similar to the one observed previously for total plasma cfDNA.^5^ The sEV-rich fraction contains a majority of small (∼150–180 bp) and also large DNA (>1,000 bp), the small DNA exhibiting similar biological properties to plasma cfDNA. This suggests that a fraction of cfDNA in the bloodstream of patients with lung cancer is associated with sEV (either inside or bound to sEV).

Prior reports indicate that tumorigenic mutations could be more easily detected in exosomes or EV from plasma samples than in cfDNA.^15^^,^^16^^,^^24^^,^^25^^,^^26^ The observed variation in the ctDNA fractions and detection rates reported in prior works can be explained by the differences in the EV isolation methods, non-standardized EV characterization post-fractionation, the diversity of methods used for detecting tumor signal, or issues related to the stochastic noise for detecting molecules with mutations in low DNA concentrations. Here, by selecting blood plasma samples from cancer samples with previously known high tumor fraction and high concentration in DNA, as well as by using state-of-the-art EV fractionation and characterization methods, and genome-wide DNA sequencing method, we by-passed these sources of potential biases. We observed that on a genome-wide scale there is no enrichment in tumor signal in the DNA associated to sEV-rich fractions, in comparison to EV-poor fractions or non-fractionated plasma cfDNA. An analysis of the copy-number aberrations from the sWGS indicate no significant differences between the copy-number aberration profile of cfDNA and EV-rich or EV-poor fractions, suggesting a similar origin for the DNA in these different samples. We also observe a relative enrichment in mtDNA in the sEV fractions, suggesting some form of protection of mitochondria and/or mtDNA by EVs in the blood.^27^

Our conclusions are limited by a relatively small cohort of lung cancer plasma samples included in this study (*n* = 16). Samples had in general a high tumor fraction (>5%) clearly detectable using sWGS. We focused our analysis to lung cancer plasma samples and cannot conclude that the tumor content in EV-DNA might differ in other cancer types or stages. The tumor content greatly differs in cfDNA from various cancer types,^28^ and we could anticipate similar differences for EV-DNA, and even more heterogeneity depending on the EV type. Finally, the genome-wide content of EV-DNA might differ depending on the fractionation method due to the diversity of EV.^29^ In particular methods based on SEC (like AFC) have the advantage to recover purer fractions of EV but are biased toward smaller EV.^30^ A study by Vagner and colleagues suggests that a subpopulation of large EVs is enriched for tumor DNA. This observation was based on a different, more complex EV purification and enrichment approach that may or may not be suited for implementation in daily clinical practice.^15^ Determining the most suitable EV isolation protocol for tumor DNA content measurements will be important for translational applications. In this study, we found that SEC-based plasma EV purification does not increase sensitivity of tumor DNA detection. In addition, we performed DNAse and/or Triton X-100 treatment of the plasma fractions, which suggest that most cfDNA is concentrated outside rather than within small EVs.^31^ The DNA amount inside small EVs is below the practical limit for DNA sequencing applications. It has been shown recently that biological cell-derived EVs can be subject to corona formation.^32^ Neuberger et al. used DNase I and proteinase K digestion to determine the amount of DNA, associated with the EV surface, or enclosed into the vesicular lumen. Their results are in concordance with our observation that only a minute part of cfDNA is associated with sEVs in human plasma.^33^ sEV-associated DNA seems largely associated with the surface as part of the corona and only found in trace amounts in the EV lumen. However, we cannot exclude that the EV-DNA in the lumen could be more concentrated in larger much less abundant EV subtypes.

In summary, cfDNA in plasma can be of dual origin, either bound to proteins (including the nucleosome, as canonically reported) and also associated to EVs. DNA associated to small EV (including exosomes) does not appear preferentially enriched in tumor signal.

### Limitations of the study

Our conclusions are limited by the overall size of our cohort, the focus on one cancer type (lung cancer) and late-stage disease. Even if there is no strong rationale to assume our conclusions could be drastically different in a different cancer type, or with early-stage cancer, this cannot be concluded based on this study.

The central question of our work was whether using the same state of art and standardizable sequencing approach (low-coverage WGS) we can compare the genomic content of cell-free DNA with EV DNA. Due to the lack of specificity of EV isolations, we selected an approach that has been demonstrated in the literature to isolate small EV with a throughput compatible with clinical use. Other EV isolation methods (e.g., ultracentrifugation or derivatives) can be more selective for EV subpopulations but present issues related to their physics and have a throughput and robustness difficult to be compatible with a “real-world” clinical use.

We observed the presence of long DNA (>1,000 bp) in one of the EV fractions. Even if our analysis using genome-wide sequencing and *in silico* size selection does not seem to confirm an enrichment in tumor signal in these long fragments, a confirmation with long-read sequencing will be needed.

Even if the main result is negative (from an EV-research focused perspective), it clearly indicates that the extra refinement needed to extract specifically, and finely, DNA from EV would drastically increase the cost and complexity of DNA analysis to reach an advantage in terms of specificity of analysis against a routine cfDNA sequencing.

Due to the lack of specificity of the SEC, it is possible that the sEV fractions contain lipoproteins (LPPs). This does not alter our conclusions that the relative tumor fraction and absolute number of DNA molecules are lower in the SEC fraction 1–5 compared to a bulk cfDNA analysis.

Plasma processing in a clinical setting include at least one step of freezing due to the storage of plasma samples after processing. This freezing step, as well as the centrifugation protocol to isolate plasma, are necessary from a logistical point of view, but might lead to the artifactual release of EV from platelets. Platelet EVs can be a potential confounding factor in the characterization and quantitation of EVs and might release genomic DNA (gDNA) in the bloodstream. We do not detect gDNA in our EV fractions, suggesting that such contaminating risk is likely to not affect our conclusions based on DNA sequencing but could impact the analysis of other circulating analytes from the EV fractions. More research will be needed to improve our understanding of the nature and biogenesis of platelets, EVs, cfDNA, and cell-free RNA.

## Resource availability

### Lead contact

Further information and requests should be directed to the lead contact, Florent Mouliere (florent.mouliere@cruk.manchester.ac.uk).

### Materials availability

This study did not generate new unique reagents.

### Data and code availability

Sequencing data was deposited to the EGA and will be available under accession number EGA00002312671. We have submitted all relevant data of our experiments to the EV-TRACK knowledgebase (EV-TRACK ID: EV240014).^30^ This paper does not report new original code. Any additional information required to reanalyze the data reported in this paper is available from the lead contact upon request.

## Acknowledgments

The authors are thankful to the Amsterdam UMC Liquid Biopsy Center for the logistical support and advices. Y.P. and F.M. are funded by the Amsterdam UMC Liquid Biopsy Center, an initiative made possible through the Stichting Cancer Center Amsterdam. N.M. and F.M. are supported by a Dutch Cancer Fund (KWF-12822). The EM at the VU campus is supported by 10.13039/501100003246NWO (91111009). This work was carried out on the Dutch national e-infrastructure with the support of SURF Cooperative.

## Author contributions

Conceptualization, F.M.; data curation, N.M. and F.M.; formal analysis, N.M. and F.M.; funding acquisition, D.M.P. and F.M.; investigation, N.M., S.V., Y.P., L.B., and J.W.; methodology, N.M., S.V., Y.P., L.B., and F.M.; project administration, F.M.; resources, I.B., D.M.P., and F.M.; software, N.M., Y.P., and F.M.; supervision, D.M.P. and F.M.; validation, N.M. and F.M.; visualization, F.M.; writing—original draft, N.M. and F.M.; writing—review & editing, N.M., S.V., Y.P., L.B., J.W., I.B., D.M.P., and F.M.

## Declaration of interests

F.M. is coinventor on patents related to cfDNA fragmentation analysis. F.M. has consulted for Roche Dx. D.M.P. is co-founder and CSO of ExBiome BV. Other coauthors have no relevant conflict of interests.

## STAR★Methods

### Key resources table

**Table:** 

REAGENT or RESOURCE	SOURCE	IDENTIFIER
**Biological samples**		

Plasma samples	Liquid Biopsy Center Amsterdam	http://www.liquidbiopsycenter.nl/

**Critical commercial assays**		

ThruPLEX Plasma-Seq	Takara	Cat #: R400492
qEVoriginal 70 nm	Izon	#SP-1

**Deposited data**		

Sequencing data	EGA	EGA00002312671

**Software and algorithms**		

FrEIA tool	Moldovan et al., 2024^34^	Github: https://github.com/mouliere-lab/FrEIA.git
ichorCNA	Adalsteinsson et al., 2017^18^	Github: https://github.com/broadinstitute/ichorCNA

### Experimental model and study participant details

Collection of pre-treatment blood samples in non-small cell lung cancer patients (*n* = 16) was conducted according to the Declaration of Helsinki and approved by the Amsterdam University Medical Center ethical committee (Table S1). Written informed consent was obtained from all participants. Gender, age, smoker status, and cancer stage and type are reported when available in Table S1. No information about ancestry, race, ethnicity, or socioeconomic status was collected for any of the participants.

### Method detail

#### Sample collection

Blood was collected in EDTA K2 tubes (BD vacutainer) and plasma isolated within 4 h. Briefly, blood was centrifuged at 900 g for 7 min at room temperature. Supernatant was carefully collected and centrifuged further at 2500 g for 10 min, aliquoted into 0.5 mL tubes and stored at −80°C.

#### EV isolation

1 mL of plasma per sample was loaded into qEV columns (iZON) with 0.5 mL PBS. This was repeated 3 times, inputting a total of 3 mL plasma per patient. Between each loading the columns were washed using 30 mL of washing buffer (PBS 1X). 20 fractions were further collected using an Izon Automated Fraction Collector (AFC) following the constructor recommendations (Table S2). AFC fractions 1 to 5, 7 to 11, 12 to 15, 16 to 20 were pooled for further analysis.

#### Electron microscopy

EV fractions were spotted on carbon/formvar-coated mesh grids. After blotting off the excess liquid, the samples were contrasted by 2% uranylacetate (Polysciences Inc, Cat No 21447-25) in water for 1 min, the excess stain was blotted off and grids were air-dried. Vesicular structures were imaged with 80 kV Tecnai 12 (ThermoFisher) TEM at 60,000× magnification using a 2k × 2k pixel CCD side-mounted camera (Veleta, EMsis GmbH).

#### Vesicle sizes distribution and concentration

The concentration and diameter of circulating vesicles were determined by tunable-resistive pulse sensing (TRPS), using an Exoid device (iZON). Samples were diluted 50× in an electrolyte buffer and analyzed using an NP250 nanopore at the average result of pressures of 300, 500, and 800 Pa. Concentration and particle size of each pool was determined by measuring calibration beads with a known diameter in nm and concentration in particle/mL and analyzed using the iZON control suite software (v. 1.0.2.32).

#### Western blot

Equal volumes of pooled AFC fractions were mixed with sample buffer (for CD63 and CD81 without reducing agent). After boiling for 10 min at 95°C, samples were loaded on a 4–15% mini-Protean TGX gel (Bio-RAD). After electrophoresis, proteins were transferred to a 0.2 μm nitrocellulose membrane (Amersham) and blocked for 30 min with 5% milk in TBS 0.1% Tween at room temperature. Primary antibody incubation was done overnight at 4°C with 5% milk in TBS 0.1% Tween followed by 1 h incubation with secondary antibody in 5% milk. Membranes were developed using Pierce ECL western blotting substrate and imaged on a Bio-Rad Gel-Doc XR + Imager. Primary antibodies used for western blot are anti-CD63 (BD Pharmingen #556019, clone H5C6), anti-CD81 (BD Pharmingen #555675, clone JS-81), anti-Alix (Cell Signaling #2171, clone #3A9), anti-TSG101 (Genetex #GTX70255, clone 4A10), anti-Flotilin 1 (Cell Signaling #18634, clone D32VJ7), anti-Calnexin (Millipore #AB2301). Secondary antibodies used were HRP conjugated rabbit-*anti*-Mouse IgG (Dako #P260) and HRP conjugated anti-rabbit IgG (Cell Signaling #7074).

#### DNA isolation

Plasma cfDNA was isolated from all samples using the QIAsymphony Circulating DNA Kit (Qiagen). 3.2 mL of plasma was used for the cfDNA extraction. DNA from EV fractions were isolated using the same protocol. The cfDNA concentration was determined using an Agilent 4200 TapeStation System with the Cell-free DNA ScreenTape Analysis assay (Agilent).

#### Treatment experiment

Fractions from 4 samples were subjected to a combination of treatments to evaluate the DNA content of the EV fractions. Protocol was adapted from a previous publication.^33^ In brief, fractions were subjected to treatment using either DNAse1 (NEB), Triton X-100 or a combination of both as previously described. Mock controls were treated using similar volume of PBS 1×. The samples were pre-incubated with Triton X-100 (0.5% final). All sample were incubated for 30 min at 37°C, shaking at 250 rpm. DNaseI (NEB) at a final concentration of 1 mg/mL and 10 × DNase reaction buffer were added and the samples were incubated for 40 min at 37°C with shaking. DNA was then isolated as described above.

#### Sequencing preparation

Sequencing libraries were prepared using 1–10 ng of cfDNA and the ThruPLEX Plasma-seq Kit (Takara Bio) according to the manufacturer’s instructions. Libraries quality was controlled using an Agilent 4200 TapeStation System with the D1000 ScreenTape Analysis assay (Agilent). Libraries were pooled on equimolar amounts and sequenced using 150 bp paired-end runs on the Illumina NovaSeq 6000 using S4 flow-cells (Illumina).

#### Whole genome sequencing analysis

Quality control of demultiplexed data was performed using the FastQC software (v0.11.9). Trimming was done using BBDuk (BBmap v38.79). Quality check post trimming was carried out with FastQC (v0.11.9). Trimmed reads were aligned to the human reference genome GRCh38 with BWA-MEM (v0.7.17) using default settings. MarkDuplicates (Picard Tools v2.22.2) was applied to annotate duplicate reads. Reads with a MAPQ score below 5, PCR duplicates, secondary alignments, supplementary alignments and unmapped reads were removed prior to further downstream analysis using samtools (v1.9). Samtools-flagstat software (v1.9) and qualimap software (v2.2.2) were used for post alignment quality check. The proportion of mtDNA was calculated using the formula [mtDNA_fraction=(reads chrM/reads aligned)∗100], where the amount of chrM reads and of aligned reads were generated using samtools (v1.9).

#### Copy number analysis

The ichorCNA software (commit 5bfc03e) was used to perform the copy number analysis and estimate the ctDNA tumor fraction.^18^ Exceptions to the software’s default settings were as follows: i) An in-house panel of shallow Whole Genome Sequencing (sWGS) normals was created, ii) non-tumor fraction parameter restart values were increased to c(0.95,0.99,0.995,0.999), iii) ichorCNA ploidy parameter restart value was set to 2, iv) no states were used for subclonal copy number and v) the maximum copy number to use was lowered to 3. The reported tumor fraction was retrieved from the data using the highest log likelihood solution.

#### cfDNA fragment size distribution analysis

The Picard InsertSizeMetrics software (v2.22.2) was used with the default settings on the mapped reads to extract cfDNA fragment size distribution.^31^ The proportion of fragments between 20 and 150 bp (P20_150) were calculated from sWGS data for comparison purposes. Plots were constructed in R (v3.6) using the packages ggplot (v3.3.5), dplyr (v1.0.7), tidyr (v1.1.3).

#### Fragment end sequence composition and diversity

Fragment ends were analyzed using the FrEIA toolkit developed in our group [https://github.com/mouliere-lab/FrEIA.git] (commit 8ecb58b) controlled by Snakemake (v. 5.14.0). In brief, trimmed and quality-filtered reads were passed to a custom pysam (v. 0.16.0.1) implementation, extracting the first 3 mapped bases from the 5′ end of the reads. Fragments were characterized based on their first mapping nucleotide and their first three mapping nucleotides. Fractions of these fragment categories were calulated for each sample.

The diversity of the 5′ trinucleotide fragment-end sequences was computed using the Gini index, with the formula:

$$G=1-\sum_{i=1}^{64}P_{i}^{2}\text{,}$$

where *P*_*i*_ is the fraction of a given *i* fragment-end sequence.

### Quantification and statistical analysis

All statistics were calculated in R (v3.6) with the package ggpubr (v0.4.0). *p* values and statistical test name were included when appropriate in the manuscript and figure captions.

## Supplemental information

Document S1. Figures S1–S6

Table S1. Patients demographic information, related to Figure 1

Table S2. Plasma samples and fractions details, related to STAR Methods

## References

[1] Heitzer E., Haque I.S., Roberts C.E.S., Speicher M.R. (2019). Current and Future Perspectives of Liquid Biopsies in Genomics-Driven Oncology. Nature Rev. Genet..

[2] van der Pol Y., Mouliere F. (2019). Toward the Early Detection of Cancer by Decoding the Epigenetic and Environmental Fingerprints of Cell-Free DNA. Cancer Cell.

[3] Wan J.C.M., Massie C., Garcia-Corbacho J., Mouliere F., Brenton J.D., Caldas C., Pacey S., Baird R., Rosenfeld N. (2017). Liquid Biopsies Come of Age: Towards Implementation of Circulating Tumour DNA. Nature Rev. Cancer.

[4] Merker J.D., Oxnard G.R., Compton C., Diehn M., Hurley P., Lazar A.J., Lindeman N., Lockwood C.M., Rai A.J., Schilsky R.L. (2018). Circulating Tumor DNA Analysis in Patients With Cancer: American Society of Clinical Oncology and College of American Pathologists Joint Review. J. Clin. Oncol..

[5] Mouliere F. (2022). A hitchhiker’s guide to cell-free DNA biology. Neurooncol. Adv..

[6] Jahr S., Hentze H., Englisch S., Hardt D., Fackelmayer F.O., Hesch R.D., Knippers R. (2001). DNA fragments in the blood plasma of cancer patients: Quantitations and evidence for their origin from apoptotic and necrotic cells. Cancer Res..

[7] Murtaza M., Dawson S.J., Pogrebniak K., Rueda O.M., Provenzano E., Grant J., Chin S.F., Tsui D.W.Y., Marass F., Gale D. (2015). Multifocal clonal evolution characterized using circulating tumour DNA in a case of metastatic breast cancer. Nat. Commun..

[8] Jeppesen D.K., Fenix A.M., Franklin J.L., Higginbotham J.N., Zhang Q., Zimmerman L.J., Liebler D.C., Ping J., Liu Q., Evans R. (2019). Reassessment of Exosome Composition. Cell.

[9] Pegtel D.M., Gould S.J. (2019). Exosomes. Annu. Rev. Biochem..

[10] Drees E.E.E., Roemer M.G.M., Groenewegen N.J., Perez-Boza J., van Eijndhoven M.A.J., Prins L.I., Verkuijlen S.A.W.M., Tran X.M., Driessen J., Zwezerijnen G.J.C. (2021). Extracellular vesicle miRNA predict FDG-PET status in patients with classical Hodgkin Lymphoma. J. Extracell. Vesicles.

[11] Yokoi A., Villar-Prados A., Oliphint P.A., Zhang J., Song X., DeHoff P., Morey R., Liu J., Roszik J., Clise-Dwyer K. (2019). Mechanisms of nuclear content loading to exosomes. Sci. Adv..

[12] Takahashi A., Okada R., Nagao K., Kawamata Y., Hanyu A., Yoshimoto S., Takasugi M., Watanabe S., Kanemaki M.T., Obuse C., Hara E. (2017). Exosomes maintain cellular homeostasis by excreting harmful DNA from cells. Nat. Commun..

[13] Thakur B.K., Zhang H., Becker A., Matei I., Huang Y., Costa-Silva B., Zheng Y., Hoshino A., Brazier H., Xiang J. (2014). Double-stranded DNA in exosomes: A novel biomarker in cancer detection. Cell Res..

[14] Allenson K., Castillo J., San Lucas F.A., Scelo G., Kim D.U., Bernard V., Davis G., Kumar T., Katz M., Overman M.J. (2017). High prevalence of mutant KRAS in circulating exosome-derived DNA from early-stage pancreatic cancer patients. Ann. Oncol..

[15] Vagner T., Spinelli C., Minciacchi V.R., Balaj L., Zandian M., Conley A., Zijlstra A., Freeman M.R., Demichelis F., De S. (2018). Large extracellular vesicles carry most of the tumour DNA circulating in prostate cancer patient plasma. J. Extracell. Vesicles.

[16] Hagey D.W., Kordes M., Görgens A., Mowoe M.O., Nordin J.Z., Moro C.F., Löhr J.M., EL Andaloussi S. (2021). Extracellular vesicles are the primary source of blood-borne tumour-derived mutant KRAS DNA early in pancreatic cancer. J. Extracell. Vesicles.

[17] Tkach M., Hego C., Michel M., Darrigues L., Pierga J.Y., Bidard F.C., Théry C., Proudhon C. (2022). Circulating extracellular vesicles provide valuable protein, but not DNA, biomarkers in metastatic breast cancer. J. Extracell. Biol..

[18] Adalsteinsson V.A., Ha G., Freeman S.S., Choudhury A.D., Stover D.G., Parsons H.A., Gydush G., Reed S.C., Rotem D., Rhoades J. (2017). Scalable whole-exome sequencing of cell-free DNA reveals high concordance with metastatic tumors. Nat. Commun..

[19] Mouliere F., Chandrananda D., Piskorz A.M., Moore E.K., Morris J., Ahlborn L.B., Mair R., Goranova T., Marass F., Heider K. (2018). Enhanced detection of circulating tumor DNA by fragment size analysis. Sci. Transl. Med..

[20] Lo Y.M.D., Han D.S.C., Jiang P., Chiu R.W.K. (2021). Epigenetics, fragmentomics, and topology of cell-free DNA in liquid biopsies. Science.

[21] Burnham P., Kim M.S., Agbor-Enoh S., Luikart H., Valantine H.A., Khush K.K., De Vlaminck I. (2016). Single-stranded DNA library preparation uncovers the origin and diversity of ultrashort cell-free DNA in plasma. Sci. Rep..

[22] Ma M.J.L., Zhang H., Jiang P., Sin S.T.K., Lam W.K.J., Cheng S.H., Lee W.S., Gai W., Tse O.Y.O., Peng W. (2019). Topologic analysis of plasma mitochondrial DNA reveals the coexistence of both linear and circular molecules. Clin. Chem..

[23] Tulkens J., De Wever O., Hendrix A. (2019). Analyzing bacterial extracellular vesicles in human body fluids by orthogonal biophysical separation and biochemical characterization. Nat. Protoc..

[24] Yu W., Hurley J., Roberts D., Chakrabortty S.K., Enderle D., Noerholm M., Breakefield X.O., Skog J.K. (2021). Exosome-based liquid biopsies in cancer: opportunities and challenges. Ann. Oncol..

[25] Castellanos-Rizaldos E., Grimm D.G., Tadigotla V., Hurley J., Healy J., Neal P.L., Sher M., Venkatesan R., Karlovich C., Raponi M. (2018). Exosome-based detection of EGFR T790M in plasma from non–small cell lung cancer patients. Clin. Cancer Res..

[26] Allenson K., Castillo J., San Lucas F.A., Scelo G., Kim D.U., Bernard V., Davis G., Kumar T., Katz M., Overman M.J. (2017). High prevalence of mutant KRAS in circulating exosome-derived DNA from early-stage pancreatic cancer patients. Ann. Oncol..

[27] van der Pol Y., Moldovan N., Ramaker J., Bootsma S., Lenos K.J., Vermeulen L., Sandhu S., Bahce I., Pegtel D.M., Wong S.Q. (2023). The landscape of cell-free mitochondrial DNA in liquid biopsy for cancer detection. Genome Biol..

[28] Bettegowda C., Sausen M., Leary R.J., Kinde I., Wang Y., Agrawal N., Bartlett B.R., Wang H., Luber B., Alani R.M. (2014). Detection of circulating tumor DNA in early- and late-stage human malignancies. Sci. Transl. Med..

[29] Xu R., Rai A., Chen M., Suwakulsiri W., Greening D.W., Simpson R.J. (2018). Extracellular Vesicles in Cancer — Implications for Future Improvements in Cancer Care. Nature Rev. Clin. Oncol..

[30] Van Deun J., Mestdagh P., Agostinis P., Akay Ö., Anand S., Anckaert J., Martinez Z.A., Baetens T., Beghein E., Bertier L. (2017). EV-TRACK: transparent reporting and centralizing knowledge in extracellular vesicle research. Nat. Methods.

[31] van der Pol Y., Moldovan N., Verkuijlen S., Ramaker J., Boers D., Onstenk W., de Rooij J., Bahce I., Pegtel D.M., Mouliere F. (2022). The Effect of Preanalytical and Physiological Variables on Cell-Free DNA Fragmentation. Clin. Chem..

[32] Wolf M., Poupardin R.W., Ebner-Peking P., Andrade A.C., Blöchl C., Obermayer A., Gomes F.G., Vari B., Maeding N., Eminger E. (2022). A functional corona around extracellular vesicles enhances angiogenesis, skin regeneration and immunomodulation. J. Extracell. Vesicles.

[33] Neuberger E.W.I., Hillen B., Mayr K., Simon P., Krämer-Albers E.M., Brahmer A. (2021). Kinetics and Topology of DNA Associated with Circulating Extracellular Vesicles Released during Exercise. Genes.

[34] Moldovan N., van der Pol Y., van den Ende T., Boers D., Verkuijlen S., Creemers A., Ramaker J., Vu T., Bootsma S., Lenos K.J. (2024). Multi-modal cell-free DNA genomic and fragmentomic patterns enhance cancer survival and recurrence analysis. Cell Rep. Med..

