# Supplementary material for: Comparison of cell-free and small extracellular-vesicle-associated DNA by sequencing plasma of lung cancer patients
Source: iScience. 2024 Aug 14;27(9):110742. doi: 10.1016/j.isci.2024.110742 (PMC11389540; doi:10.1016/j.isci.2024.110742)
Supplement: Document S1. Figures S1–S6 [file mmc1.pdf]

iScience, Volume *27*

Supplemental information

Comparison of cell-free and small extracellular-vesicle-associated DNA by sequencing plasma of lung cancer patients

Norbert Moldovan, Sandra Verkuijlen, Ymke van der Pol, Leontien Bosch, Jan R.T. van Weering, Idris Bahce, D. Michiel Pegtel, and Florent Mouliere

Supplementary materials for:

Comparison of cell-free and small extracellular vesicles DNA sequencing in the plasma of lung cancer patients.

Norbert Moldovan[1, $,] Sandra Verkuijlen[1, $,] Ymke van der Pol[1, $,] Leontien Bosch[1], Jan R.T. van Weering[2], Idris Bahce[3], D. Michiel Pegtel[1, #], Florent Mouliere[1, #]

1. Amsterdam UMC, Vrije Universiteit Amsterdam, Department of Pathology, Cancer Center Amsterdam, 1081 HV, Amsterdam, The Netherlands.
2. Amsterdam UMC, Vrije Universiteit Amsterdam, Department of Human Genetics and Functional Genomics, Center for Neurogenomics and Cognitive Research, 1081 HV, Amsterdam, The Netherlands.
3. Amsterdam UMC, Vrije Universiteit Amsterdam, Department of Pulmonology, Cancer Center Amsterdam, 1081 HV, Amsterdam, The Netherlands.

Supplementary Figures:

Supplementary Figure 1:

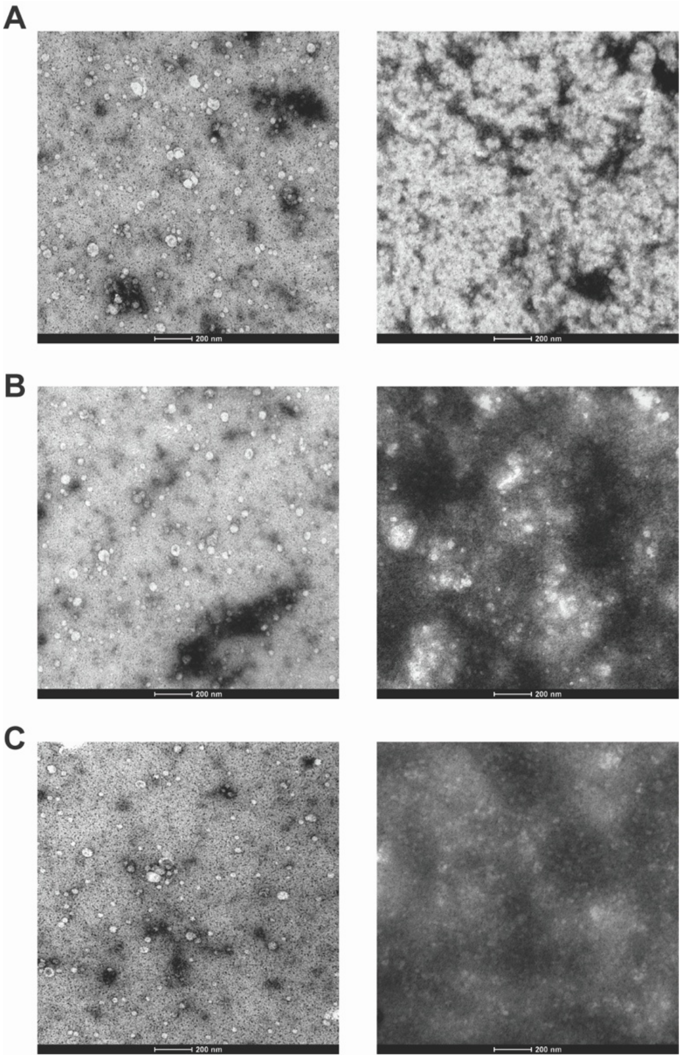

Supplementary Figure 1: EM pictures from AFC fractions 1-5 (left panels) and AFC fractions 12-15 (right panels), related to Figure 1. 10 pictures were taken per sample and one was randomly selected in this plot. Pictures were taken from 3 patients in total. Scale bar indicates 200 nm. **A:** patient Code53. **B:** patient Code148. **C:** patient Code308.

Supplementary Figure 2:

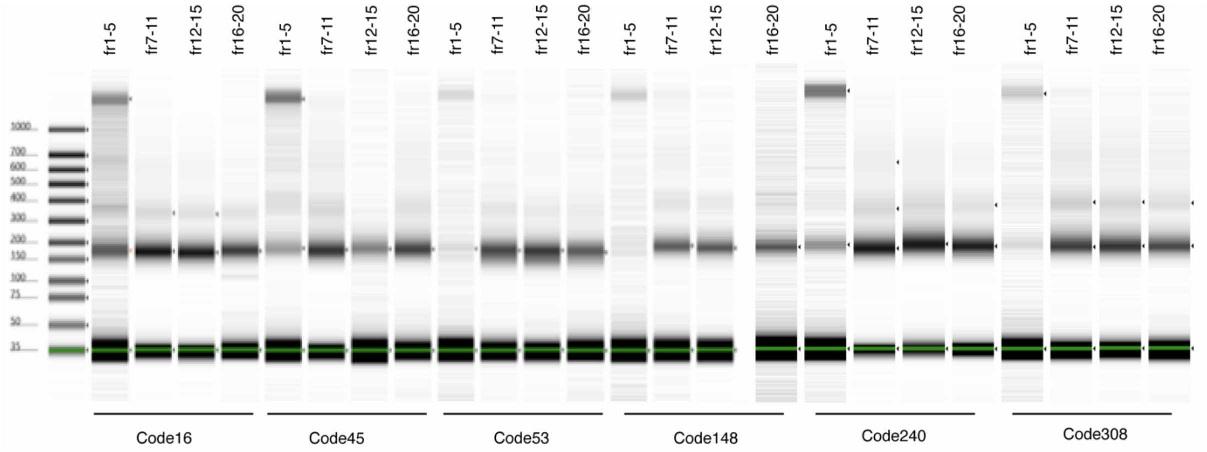

Supplementary Figure 2: DNA concentration and size profile of the different fractions measured by Tapestation, related to Figure 2..

Supplementary Figure 3:

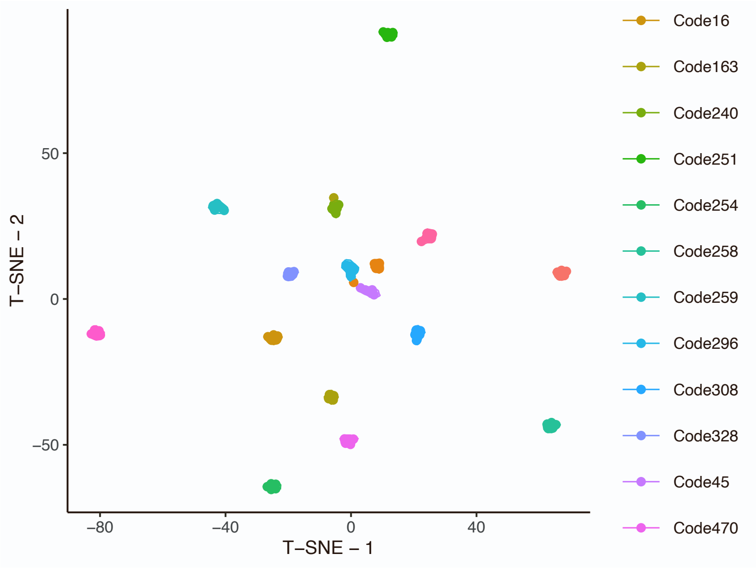

Supplementary Figure 3: t-SNE clustering of the $\log_2$ratio copy number aberration profiles obtained from the sequencing data, related to Figure 3. The colors indicate the cfDNA and EV fractions samples originating from the same patient.

Supplementary Figure 4:

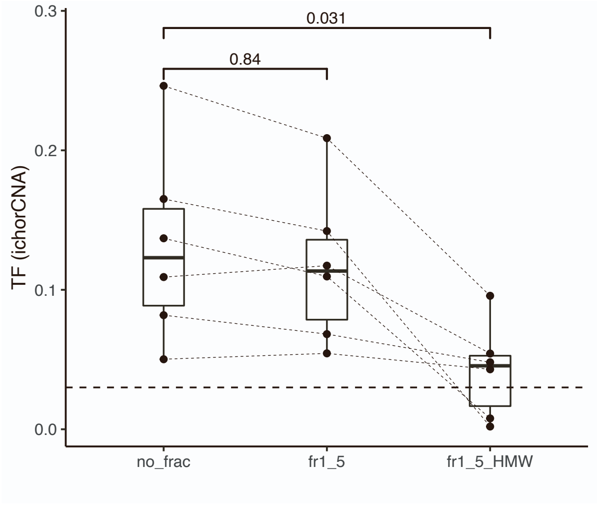

Supplementary Figure 4: tumor fraction calculated from the SCNA data in cfDNA, short and long EV DNA, related to Figure 3. Tumor fractions are determined using ichorCNA. Fr1_5_HMW: long EV DNA (>1000 bp) recovered from the AFC fraction 1-5. P values are indicated (paired Wilcoxon test).

Supplementary Figure 5:

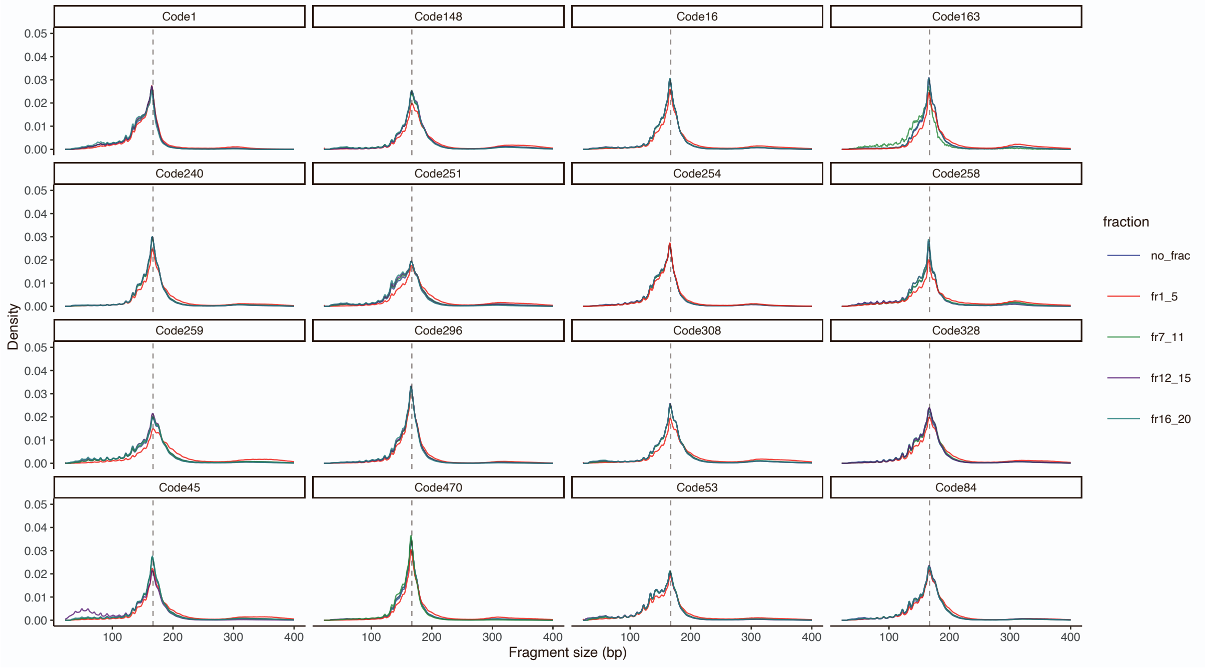

Supplementary Figure 5: fragment size distribution of the cfDNA unfractionated samples (no_frac) and AFC samples (fr1_5; fr7_11; fr12_15; fr16_20), related to Figure 4. Fragment sizes are recovered using paired-end lcWGS data.

Supplementary Figure 6:

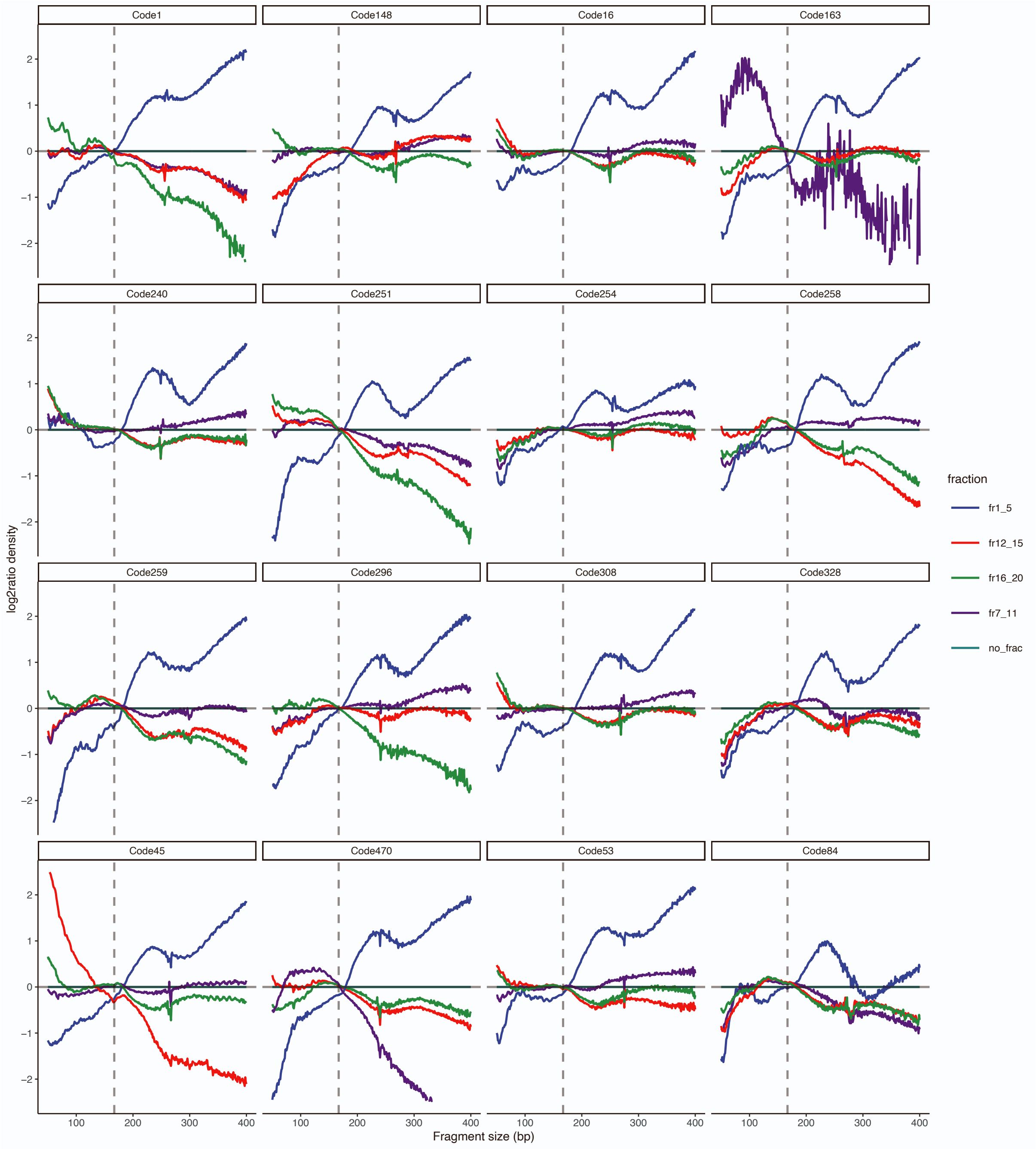

Supplementary Figure 6: $\log_2$ratio of the fragment size distribution comparing the AFC samples (fr1_5; fr7_11; fr12_15; fr16_20) to the cfDNA size profile (no_frac), related to Figure 4. Fragment sizes are recovered using paired-end lcWGS data.
